# Case Report: A giant dedifferentiated liposarcoma in the retroperitoneum

**DOI:** 10.3389/fonc.2026.1751424

**Published:** 2026-04-22

**Authors:** Jinglin Xu, Linru Yang, Haoyang Zhang, Qingliang Hu, Yuan Shen, Jian Yang, Mingxu Da

**Affiliations:** 1 Lanzhou University, Lanzhou, Gansu, China; 2 Gansu University of Chinese Medicine, Lanzhou, Gansu, China; 3 Zhangxian People’s Hospital, Dingxi, Gansu, China; 4 Chengxian People’s Hospital, Longnan, Gansu, China; 5 Gansu Provincial People’s Hospital, Lanzhou, Gansu, China

**Keywords:** dedifferentiated liposarcoma, en bloc resection, fish, radiotherapy, retroperitoneal liposarcoma

## Abstract

This article presents a 60-year-old woman presented with a one-year history of abdominal distension, which acutely worsened over four days prior to admission. Imaging revealed a large retroperitoneal liposarcoma. Exploratory laparotomy identified a massive tumor (approximately 45×40×13 cm, weighing 8 kg) invading the left kidney, adrenal gland, adnexa, left hemicolon, and mesentery. En bloc resection was performed. Postoperative pathology combined with fluorescence *in situ* hybridization (FISH) testing demonstrated MDM2/CDK4 amplification, confirming dedifferentiated liposarcoma (DDLPS). The patient recovered well postoperatively and was discharged following adjuvant radiotherapy (IMRT; 50 Gy in 25 fractions), chemotherapy (AD regimen), and immunotherapy (sintilimab).Through this case, we mainly understand the characteristics of the disease onset, special diagnosis, and main treatment methods.

## 1 Introduction

Liposarcoma represents one of the most common soft tissue sarcomas in adults, accounting for approximately 20% of cases (1). The retroperitoneum is a predominant site for liposarcoma development, attributed to its unique anatomical characteristics, with a male predominance (male-to-female ratio ~2:1). Due to the spacious retroperitoneal compartment, tumors often grow insidiously to considerable size before becoming symptomatic, typically upon compression or invasion of adjacent structures (2).

According to the World Health Organization (WHO) classification, liposarcoma is categorized into the following subtypes: well-differentiated liposarcoma (WDLS), dedifferentiated liposarcoma (DDLPS), myxoid liposarcoma, and pleomorphic liposarcoma. DDLPS is characterized by an admixture of well-differentiated components and non-lipogenic, high-grade sarcomatous elements. This subtype exhibits marked invasiveness, a poor prognosis, and high recurrence rates. DDLPS has an annual incidence of 0.5 to 1.0 cases per million population and most commonly affects individuals aged 40 to 60 years.

Diagnosis of dedifferentiated liposarcoma (DDLPS) necessitates the integration of clinical presentation, imaging features, histopathological morphology, and molecular testing results. Fluorescence *in situ* hybridization (FISH) detection of MDM2 gene amplification serves as a cornerstone molecular marker for diagnosing both well-differentiated liposarcoma (WDLS) and DDLPS, while CDK4 amplification provides additional diagnostic utility (3). Surgical resection remains the mainstay of treatment, with R0 resection being paramount for patient prognosis. En bloc resection is often essential for tumors invading adjacent organs (4). Postoperative management should incorporate multimodal adjuvant strategies, including radiotherapy and chemotherapy, tailored to the individual patient’s condition (5, 6). I summarized the diagnosis, treatment, and prognosis of liposarcoma over the past 10 years, as shown in **Table 1**. The uniqueness of this report lies in the fact that the tumor is huge, the diagnostic method is special, the molecular typing is rare, and determining the treatment plan through multidisciplinary discussion can improve patient prognosis.

**Table 1:** Diagnosis, treatment, and prognosis of patients with different types of liposarcoma.

First author	Age/sex	Symptom	FISH	Preoperative diagnosis	Treatment	Prognosis
Peng-JieMa	42/F	Asymptom	MDM2(+) CDK4(+)	DDLPS	LEFT RM	No recurrence
Li-QinLiu	50/F	Chest discomfort	MDM2(+) CDK4(+)	DDLPS	Thoracotomy	No recurrence
Jian-FengZhang	53/M	Epigastric discomfort	MDM2(-)	RPLS	Laparotomy	No recurrence
Jia-LinRen	58/F	Chest discomfort	MDM2(+)	DDLPS	Thoracotomy	No recurrence
Lu-QiangCao	63/M	Chest discomfort	MDM2(+)	DDL	Thoracotomy	No recurrence
Ying-JieGao	68/F	Lumbar pain	CDK4(+)	NO	Laparotomy	No recurrence
Zhu-Shuai Diao	53/F	Epigastric discomfort	MDM2(+) CDK4(+)	ALT	Laparotomy	No recurrence
Li-Wei Fu	42/M	Asymptom	MDM2(+) CDK4(+)	DDLPS	Thoracotomy	No recurrence
Xiao-Jing Wu	52/F	Epigastric discomfort	MDM2(+) CDK4(+)	DDLPS	Tumorectomy	No recurrence
Ying-Liao	43/F	Asymptom	MDM2(+) CDK4(+)	ALT	Tumorectomy	No recurrence
Zu-ZhuangWu	65/M	Asymptom	MDM2(+) CDK4(+)	RPLS	Laparotomy	No recurrence
Ze-KangWang	49/M	Epigastric discomfort	CDK4(-)	DDLPS	DP+S	No recurrence

PET, positron emission tomography; CT, computed tomography; pNET, pancreas neuroendocrine tumor; MRI, magnetic resonance imaging; F, female; M, male; PD, pancreaticoduodenectomy; DP+S, Distal Pancreatectomy + Splenectomy; RAMPS, Radical Antegrade Modular Pancreatosplenectomy.

## 2 Case description

**Figure 1:**
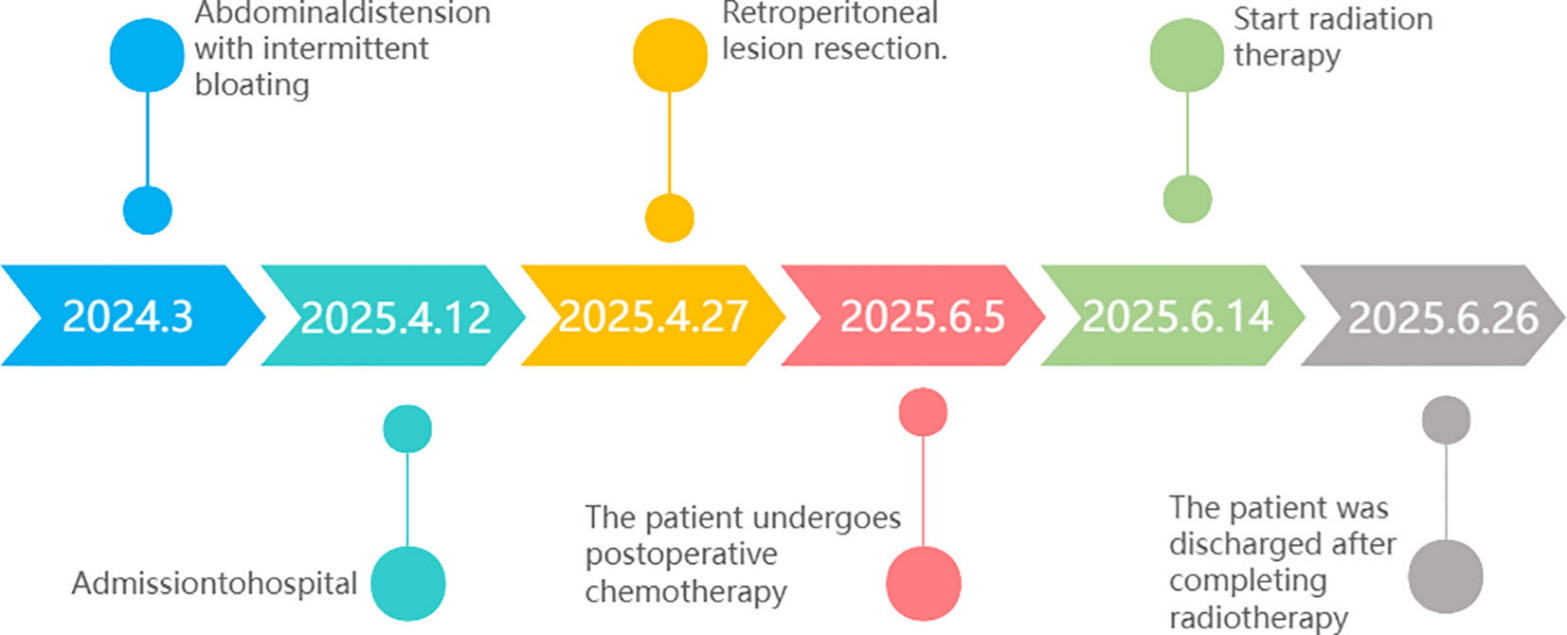
The timeline shows the onset of illness and treatment from March 2024 to June 2025.

A 60-year-old woman presented with a one-year history of abdominal distension and acute worsening of bloating over four days prior to admission. She denied abdominal pain, nausea, or vomiting. Her past medical history was unremarkable, and she reported no significant family history. Our study adhered to the Declaration of Helsinki. Ethical approval for this study was obtained from the Ethics Committee of Affiliated Hospital of Gansu Provincial People’s Hospital. The patient provided informed consent to participate in the study. The following is a timeline chart of this case report.(**Figure 1**).

### 2.1 Diagnostic journey

**Figure 2:**
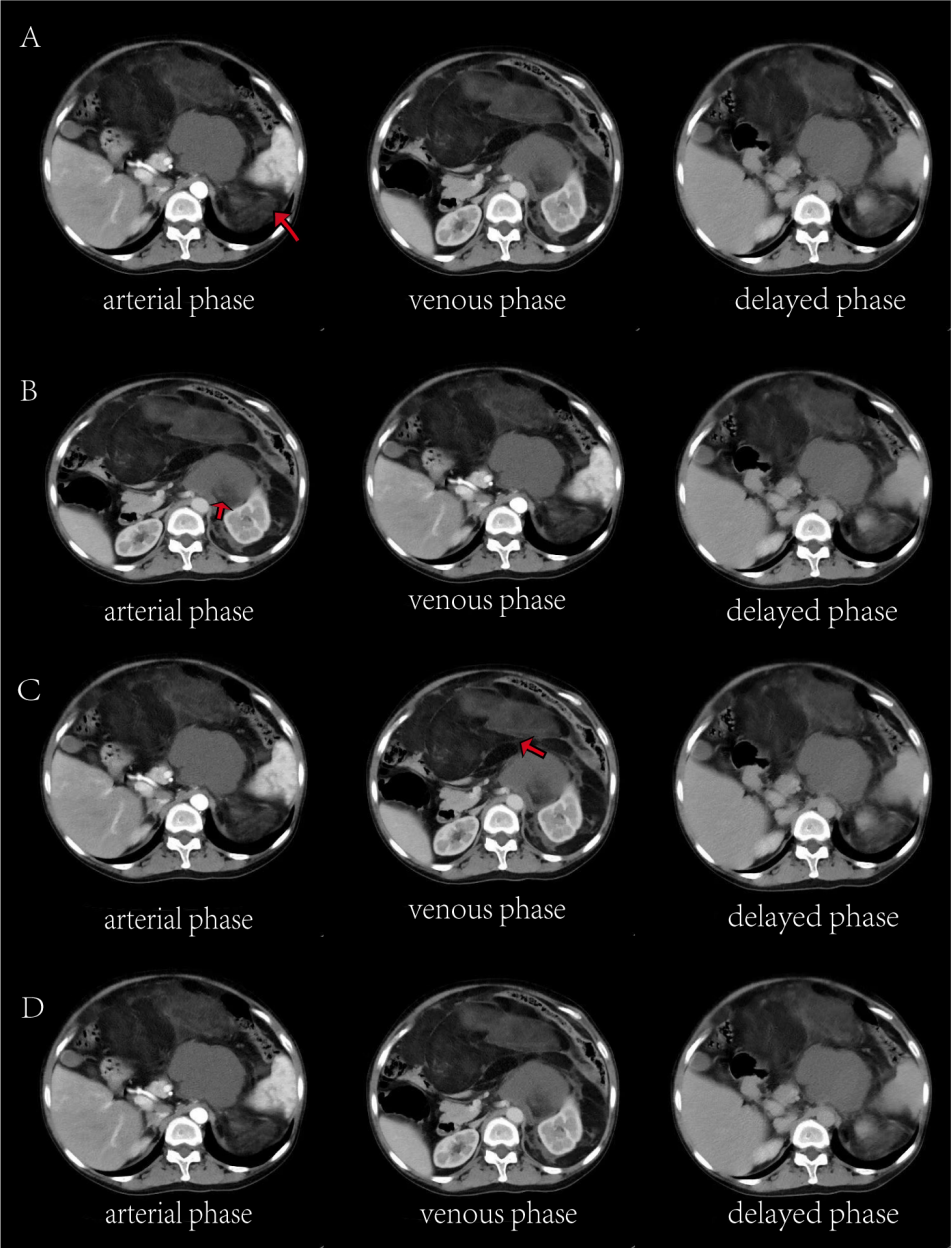
Axial CT images in the arterial **(A)**, venous **(B)**, and delayed **(C)** phases demonstrate four discrete lesions. **(A)** Arrow indicates a lesion containing hypodense fatty components. **(B)** Arrow denotes the left renal artery. **(C)** Arrow highlights a lesion with hyperdense solid components.

This patient’s abdominal examination revealed distension with a large, firm, ill-defined, poorly mobile mass partially fixed to the abdominal wall. There was no tenderness or spontaneous pain. Bowel sounds were diminished. Laboratory investigations revealed mild anemia (hemoglobin 104 g/L) and elevated inflammatory markers (interleukin-6 [IL-6]: 34.23 pg/mL). Abdominal computed tomography (CT) demonstrated a large heterogeneous retroperitoneal mass containing alternating areas of fat and soft tissue density, with significant heterogeneous enhancement, highly suggestive of liposarcoma (**Figure 2**) (7).

**Figure 3:**
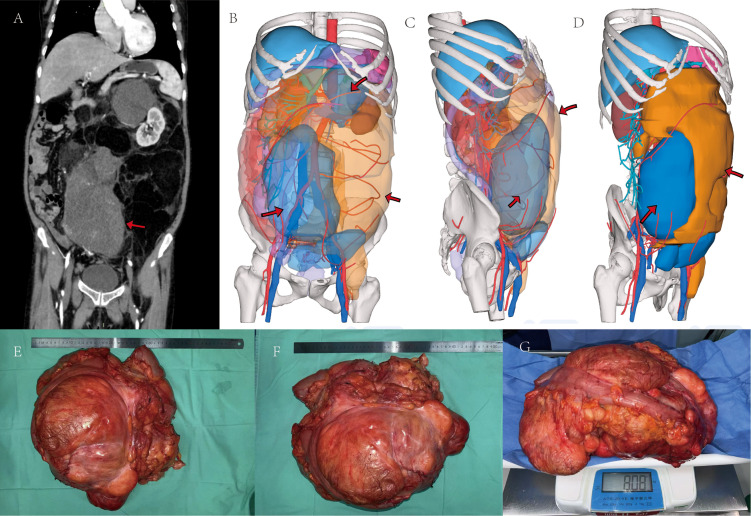
**(A)** Sagittal abdominal CT scan demonstrating a large, heterogeneous intra-abdominal mass. **(B-D)** Three-dimensional reconstructions highlighting solid (blue arrows) and fatty (yellow arrows) components. **(E-G)** Intraoperative view of the resected tumor, measuring approximately 40 × 45 cm and weighing 8.08 kg.

Following multidisciplinary team (MDT) assessment, the patient underwent exploratory laparotomy on 17 April 2025. Intraoperatively, a large retroperitoneal tumor was identified, extensively involving the left kidney, adrenal gland, ureter, adnexa, and left hemicolon (8). En bloc resection of the massive retroperitoneal tumor, incorporating the left kidney, adrenal gland, ureter, adnexa, and left hemicolon, was performed. The procedure was completed successfully without intraoperative complications, achieving satisfactory hemostasis. Postoperatively, the patient received supportive care including intravenous fluid resuscitation, antimicrobial prophylaxis, nutritional support, anemia correction, and management of hypoproteinemia. The postoperative course was uncomplicated. Gross specimen: The tumor was greyish-yellow to greyish-white, measured 45 × 40 × 13 cm, weighed approx. 8 kg, was soft in texture, and partially gelatinous (**Figure 3**).

**Figure 4:**
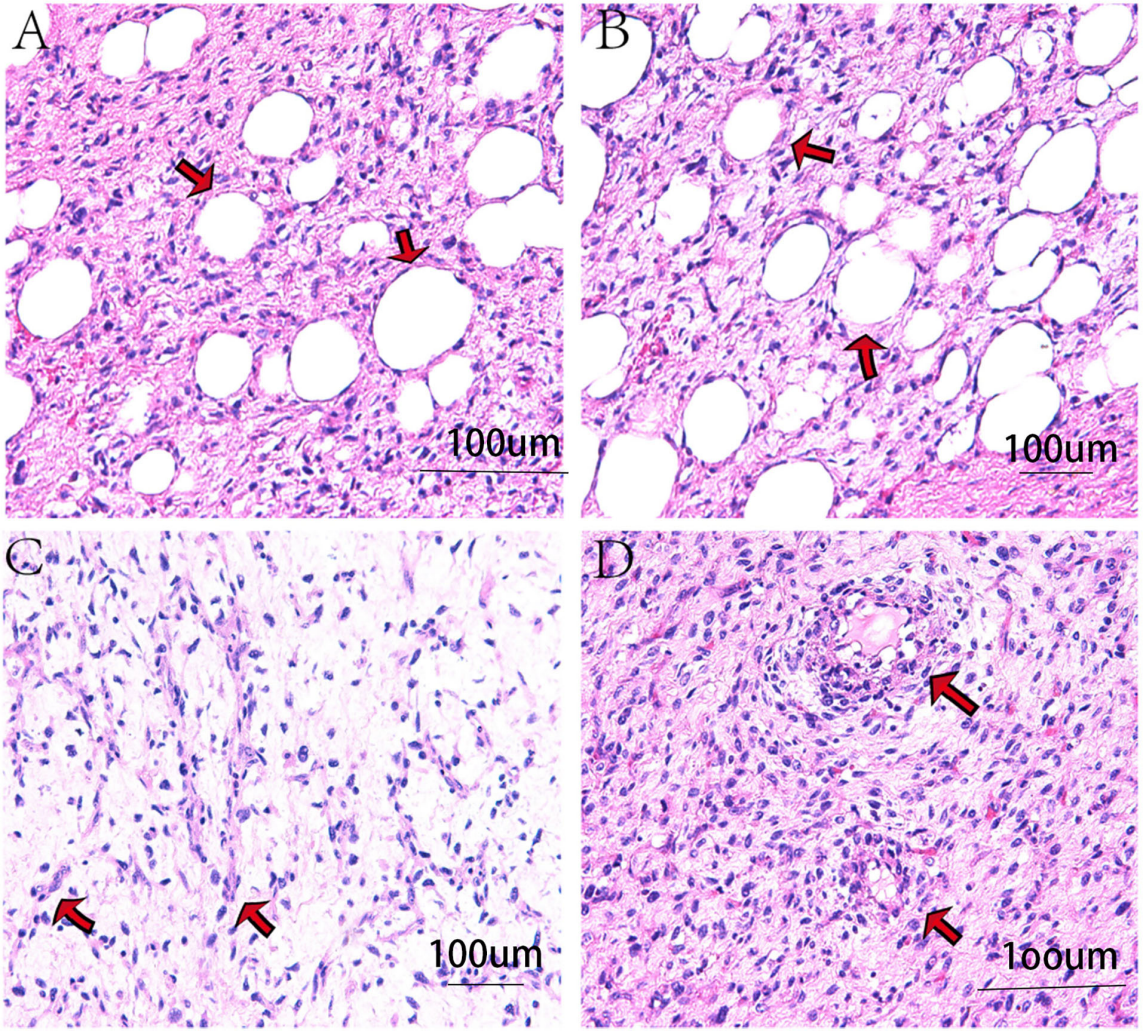
Photomicrographs show histopathological features. **(A, B)** Same field at ×100 and ×200 magnification, respectively: arrows indicate adipocytes within diffusely distributed stromal tissue. **(C, D)** Same field at ×200 and ×100 magnification, respectively: arrows highlight lipoblasts (ring-shaped cells with vacuolated cytoplasm). Diagnosis: Retroperitoneal well-differentiated liposarcoma (based on H&E staining).

Microscopically: Adipocytes were diffusely distributed, with areas of high-grade non-adipogenic sarcoma visible. (**Figure 4**).

**Figure 5:**
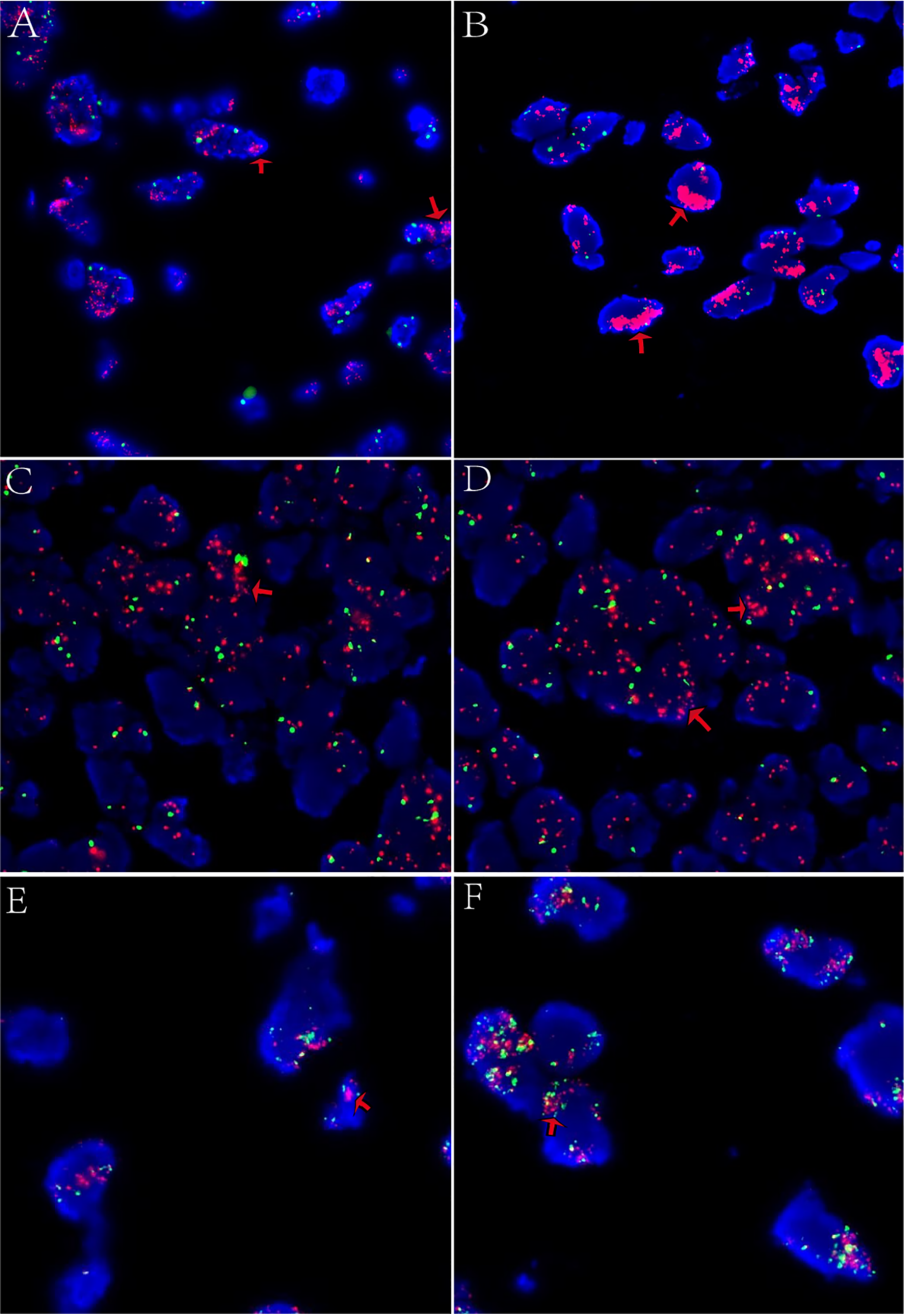
Fluorescence *in situ* hybridization (FISH) analysis. **(A, B)** MDM2 amplification (red signals; clustered distribution) in 100 tumor cells. **(C, D)** CDK4 amplification (red signals; clustered distribution). **(E, F)** DDIT3 break-apart assay: No gene rearrangement detected (negative; 100 cells scored). Clustered telomeric signal amplification indicates segmental amplification at 12q13..

**Figure 6:**
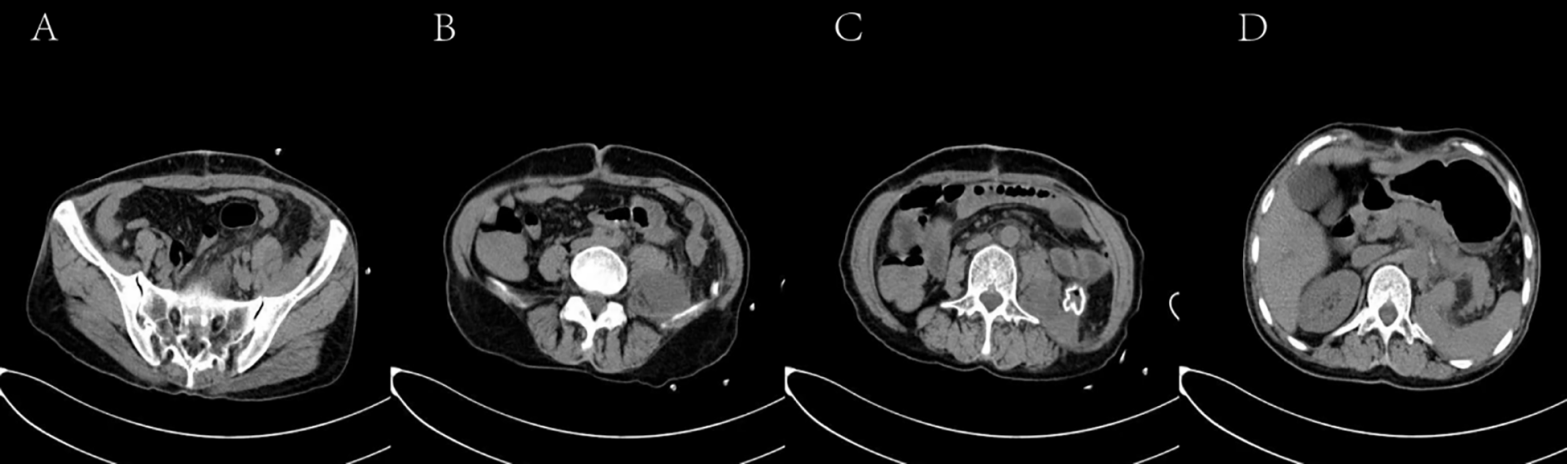
Complete macroscopic resection of the tumor.

FISH testing: MDM2 and CDK4 amplification were positive, while DDIT3 breakage was negative. (**Figure 5**) Final diagnosis: Retroperitoneal dedifferentiated liposarcoma (DDLPS). Postoperative imaging follow-up demonstrated postoperative changes in the surgical bed with no evidence of residual tumor. (**Figure 6**).

### 2.2 Follow-up

The patient initiated intensity-modulated radiation therapy (IMRT; 50 Gy in 25 fractions) (9, 10) in combination with AD chemotherapy (epirubicin plus dacarbazine) and the anti-PD-1 antibody sintilimab. Based on the STRASS trial, the routine use of postoperative radiotherapy remains controversial, but it can be considered in patients with high-risk subtypes (such as dedifferentiated tumors or positive margins) in an attempt to improve local control. In addition, since dedifferentiated subtypes of liposarcoma carry a risk of systemic dissemination, they may theoretically benefit from systemic chemotherapy. Therefore, the ongoing STRASS-2 trial, specifically targeting high-risk dedifferentiated liposarcoma, aims to evaluate the value of neoadjuvant chemotherapy (doxorubicin and ifosfamide). The prognosis of this case is of significance to this study. At the two-month postoperative follow-up, there was no evidence of recurrence or metastasis, and the patient was recovering well.

## 3 Discussion

This case involved a retroperitoneal dedifferentiated liposarcoma (DDLPS) characterized by an insidious presentation, large tumor size, and multi-organ involvement, posing significant management challenges. Successful en bloc resection was pivotal for achieving R0 margins (11). Fluorescence *in situ* hybridization (FISH) confirmed *MDM2/CDK4* gene amplification, solidifying the diagnosis and guiding subsequent targeted and systemic therapy decisions (12). While DDLPS typically exhibits poor response to conventional radiotherapy and chemotherapy, adjuvant therapy remains beneficial for reducing local recurrence in high-risk patients (13, 14). Accordingly, an individualized multimodal approach incorporating adjuvant intensity-modulated radiotherapy (IMRT), anthracycline-based chemotherapy, and immunotherapy (anti-PD-1 antibody) was implemented. Long-term surveillance is essential to monitor for recurrence and distant metastasis.

## 4 Conclusion

Retroperitoneal dedifferentiated liposarcoma (DDLPS) is characterized by an insidious onset that often eludes early detection, typically presenting at an advanced stage with substantial tumor burden. Achieving R0 resection through en bloc surgical excision, frequently requiring multivisceral resection to ensure negative margins, represents the cornerstone of curative intent. Definitive subtyping relies on fluorescence *in situ* hybridization (FISH) for *MDM2/CDK4* gene amplification. Postoperative management should incorporate adjuvant radiotherapy, systemic chemotherapy, and emerging immunotherapeutic strategies to optimize long-term outcomes. Multidisciplinary team (MDT) collaboration is indispensable throughout the diagnostic, therapeutic, and surveillance continuum.

## Data Availability

The original contributions presented in the study are included in the article/supplementary material. Further inquiries can be directed to the corresponding authors.
